# Regulation of FOXOs and p53 by SIRT1 Modulators under Oxidative Stress

**DOI:** 10.1371/journal.pone.0073875

**Published:** 2013-09-11

**Authors:** Yusuke S. Hori, Atsushi Kuno, Ryusuke Hosoda, Yoshiyuki Horio

**Affiliations:** 1 Department of Pharmacology, School of Medicine, Sapporo Medical University, Sapporo, Japan; 2 Department of Cardiovascular, Renal, and Metabolic Medicine, School of Medicine, Sapporo Medical University, Sapporo, Japan; Northwestern University Feinberg School of Medicine, United States of America

## Abstract

Excessive reactive oxygen species (ROS) induce apoptosis and are associated with various diseases and with aging. SIRT1 (sirtuin-1), an NAD+-dependent protein deacetylase, decreases ROS levels and participates in cell survival under oxidative stress conditions. SIRT1 modulates the transcription factors p53, a tumor suppressor and inducer of apoptosis, and the forkhead O (FOXO) family, both of which play roles for cell survival and cell death. In this study, we aimed to know which is working greatly among p53 and FOXOs transcription factors in SIRT1’s cell protective functions under oxidative stress conditions. The antimycin A-induced increase in ROS levels and apoptosis was enhanced by SIRT1 inhibitors nicotinamide and splitomicin, whereas it was suppressed by a SIRT1 activator, resveratrol, and a SIRT1 cofactor, NAD+. SIRT1-siRNA abolished the effects of splitomicin and resveratrol. p53-knockdown experiment in C2C12 cells and experiment using p53-deficient HCT116 cells showed that splitomicin and resveratrol modulated apoptosis by p53-dependent and p53-independent pathways. In p53-independent cell protective pathway, we found that FOXO1, FOXO3a, and FOXO4 were involved in SOD2’s upregulation by resveratrol. The knockdown of these three FOXOs by siRNAs completely abolished the SOD2 induction, ROS reduction, and anti-apoptotic function of resveratrol. Our results indicate that FOXO1, FOXO3a and FOXO4, are indispensable for SIRT1-dependent cell survival against oxidative stress, although deacetylation of p53 has also some role for cell protective function of SIRT1.

## Introduction

Reactive oxygen species (ROS) are generated as a natural byproduct of cellular metabolism. They are also produced in cells by exogenous sources, such as ionizing radiation and cytotoxic drugs. Excess amounts of ROS induce cell death, which is associated with a wide range of disorders, including cardiovascular, muscular, and neurodegenerative diseases [1]–[3]. Sirtuin-1 (SIRT1) is an NAD^+^-dependent protein deacetylase, the activation of which significantly decreases ROS levels and promotes cell survival [4]. Two important transcription factors that profoundly affect cell survival and cell death are modulated by SIRT1. One is p53, a tumor suppressor protein called the “guardian of the genome,” because of its role in preventing mutations. Irreparable DNA damage by ROS leads to the stabilization and activation of p53 [5], resulting in the expression of pro-apoptotic proteins such as BAX and PUMA, which eventually target the mitochondria and induce apoptosis [6]. The deacetylation of p53 by SIRT1 inhibits p53’s oxidative stress-induced apoptotic activity [7], [8].

Other targets of SIRT are the forkhead box O (FOXO) transcription factors [9]. Similar to p53, the FOXOs (FOXO1, FOXO3a, and FOXO4) are conserved from Drosophila to humans and induce apoptosis by up-regulating Fas, TRAIL, and Bim upon cellular stress [9]. In contrast to their promotion of apoptosis, FOXOs are also important for cell survival, by transactivating ROS-detoxifying enzymes such as superoxide dismutase 2 (SOD2/MnSOD) and catalase [9]. Therefore, FOXOs have dual roles in ROS-induced cell death and survival. The effects of SIRT1 on the FOXOs’ functions are complex and vary depending on the FOXO target genes. SIRT1 promotes the expression of FOXO target genes involved in stress resistance, while decreasing the transcription of genes involved in apoptosis [10]. Thus, SIRT1 appears to shift the FOXOs-dependent response away from cell death and toward stress resistance.

Resveratrol (3,5,4′-trihydroxy-*trans*-stilbene) (RSV), an anti-oxidative polyphenol found in grapes, red wine, berries, knotweed, peanuts, and other plants, activates SIRT1 [11] and may mediate the cardioprotective effects of red wine [12]. RSV administration exerts beneficial effects against cardiovascular and neurodegenerative diseases [4]. We previously showed that RSV retards the progression of chronic heart failure in cardiomyopathic TO2 hamsters [13] and suppresses the skeletal and cardiac muscle pathologies in dystrophin-deficient *mdx* mice, a model of Duchenne muscular dystrophy [14], [15]. Such beneficial effects are thought to be at least partly attributable to the increased SIRT1 activity [11], [13]–[15]. Although RSV itself is an anti-oxidant, SIRT1 knockdown prevents RSV’s ROS-reducing and anti-apoptotic activities in C2C12 myoblast cells, indicating that SIRT1 mediates RSV’s cell survival-promoting effects [13], [14], [16]. In C2C12 cells, RSV increases the SOD2 levels and inhibits ROS-dependent apoptosis via SIRT1 [13], whereas SIRT1 knockdown increases the levels of NADPH oxidase (NOX) family members, which are membrane proteins that generate O_2_^−^ [14]. In fact, RSV administration increases the SOD2 level in the cardiomyocytes of TO-2 hamsters [13] and decreases the NOX family mRNAs in the skeletal muscle of *mdx* mice [14]. These results indicate that SIRT1 affects cellular ROS levels and cell survival via multiple pathways; however, how p53 and FOXOs participate in the SIRT1 signaling remains to be elucidated.

In this study, we focused on the roles of p53 and FOXOs in the anti-oxidative and anti-apoptotic function of SIRT1 in C2C12 cells treated with antimycin A, which increases and releases ROS from mitochondria by inhibiting mitochondrial respiratory chain complex III. We show that modulators of SIRT1 profoundly affected the cellular ROS levels and cell survival under oxidative stress. Whereas p53 was partly involved in the antimycin A-induced apoptosis of C2C12 cells, the knockdown of three members of the FOXO family, FOXO1, FOXO3a, and FOXO4, completely abolished RSV’s ROS-reducing and anti-apoptotic activities. These FOXOs contributed to SOD2’s induction by RSV. Thus, FOXO1, FOXO3a, and FOXO4 are indispensable for RSV’s ROS-reducing and anti-apoptotic activities in C2C12 cells.

## Materials and Methods

### Cell Culture and Treatment

C2C12 mouse myoblasts (ATCC) were cultured in Dulbecco’s modified Eagle’s medium (Wako Pure Chemical Ind., Osaka, Japan) supplemented with 1% antibiotic-antimycotic mixed stock solution (Nacalai Tesque, Kyoto Japan) and 10% fetal bovine serum (MP Biomedicals, Aurora, OH, USA). HCT116 cells [17] were gifted by Dr. T. Tokino (Sapporo Medical University). Cells were pretreated with 10 or 30 µM RSV for 3 hrs followed by the addition of antimycin A at the indicated doses for 24 hrs. In experiments to analyze acetylation of p53 and FOXO protein, cell treatment was carried out in the presence of trichostatin A (TSA, 50 nM).

### Reagents and Antibodies

NAD^+^ (Oriental Yeast, Tokyo, Japan) and nicotinamide (Wako Pure Chemical Ind.) were dissolved in culture medium. Resveratrol (Wako Pure Chemical Ind.), splitomicin (Calbiochem-Millipore, Billerica, MA, USA), Ex527 (Tocris Bioscience, Ellisville, MO, USA), and antimycin A (Sigma Aldrich, St Louis, MO, USA) were dissolved in dimethyl sulfoxide to obtain stock solutions. H_2_O_2_ and Hoechst 33342 were purchased from Wako Pure Chemical Ind. The antibodies used were anti-active caspase 3 rabbit polyclonal (Abcam, Cambridge, MA, USA), anti-SOD2 rabbit polyclonal (Millipore), anti-acetyl-p53 rabbit polyclonal (detecting acetyl-Lys 379 on mouse p53 and acetyl-Lys 382 on human p53), anti-p53 mouse monoclonal, anti-FOXO1 rabbit monoclonal, anti-FOXO3a rabbit monoclonal, anti-phospho-AMPKα (Thr172) rabbit polyclonal, anti-AMPKα rabbit polyclonal, anti-phospho-acetyl-CoA carboxylase (ACC) (Ser79) rabbit polyclonal, anti-ACC rabbit polyclonal (Cell Signaling Technology, Tokyo, Japan), anti-acetyl-FOXO1 rabbit polyclonal, anti-FOXO4 goat polyclonal, anti-Bax mouse monoclonal (Santa Cruz), anti-GAPDH mouse monoclonal (Sigma Aldrich), and anti-SIRT1 rabbit polyclonal [18] antibodies.

### siRNA Transfection

siRNA against mouse SIRT1 (siTrio) was obtained from B-Bridge International as described previously [13]. siRNAs against mouse FOXO1, FOXO3a, and FOXO4 were obtained from Santa Cruz. A nonsense siRNA obtained from Sigma Genosys was used as the control for nonspecific effects on gene expression, as described previously [13], [14], [16]. Transfection was performed using a Nucleofector kit (Lonza, Walkersville). siRNAs (100 nM) were transfected twice into cells with an interval of 24 hours. Twenty-four hours after the second transfection, the cells were treated with antimycin A.

### Analysis of Cell Death

Apoptotic cell death was detected by nuclear condensation or by the immunostaining of active caspase-3 or Bax. Cells attached to glass slides were fixed with 4% paraformaldehyde for 10 min after treatment with antimycin A or H_2_O_2_. To detect apoptosis as nuclear condensation, the cells were washed with PBS followed by incubation with Hoechst 33342 (1∶1000 dilution) for nuclear staining. Immunostaining was performed as described below. The percentage of cells with condensed nuclei or of active caspase-3-positive, or Bax-positive cells was determined from six fields of each treatment, and the data from three independent experiments were combined and analyzed. Dead cells were also analyzed by the Muse™ Count and Viability Kit (Muse™ Cell Analyzer, Millipore) according to the manufacturer’s protocol.

### Analysis of Intracellular ROS Levels

Intracellular ROS levels were monitored by the confocal laser microscopic analysis of cells stained with 5-(and-6)-chloromethyl-2′,7′-dichlorodihydrofluorescein diacetate acetyl ester (CM-H_2_DCFDA) (Invitrogen). Cells attached to glass slides were incubated with CM-H_2_DCFDA for 30 min at 37°C followed by two washes with PBS. After being treated with antimycin A or H_2_O_2_, the cells were fixed with 4% paraformaldehyde for 10 min, and washed with PBS. All images were captured under the same conditions, and the fluorescence was quantified by Image-J Software (NIH). The average fluorescence intensity was obtained from six fields for each treatment, and the data from three independent experiments were combined and analyzed.

### Immunostaining

After fixation and washing, cells were blocked with PBS containing 3% BSA, 1% goat serum, and 0.1% Triton X-100 for 30 min. The cells were then incubated with antibodies against active caspase-3 (1∶250 dilution), SOD2 (1∶500 dilution), Bax (1∶50 dilution), or acetyl-p53 (1∶250 dilution) overnight at 4°C. The cells were then washed four times with PBS and incubated with secondary antibodies, Alexa Fluor 488 or 594 anti-rabbit IgG (Invitrogen, 1∶1000 dilution) overnight at 4°C. After being washed with PBS, the cells were stained with Hoechst 33342 and mounted on glass slides. During confocal microscopic observation, all the images were taken using the same settings.

### Western Blotting

Cells were lysed in CelLytic M Cell Lysis Reagent (Sigma) with 1% protease inhibitor cocktail (Nacalai Tesque). For analyses of acetyl-p53 or acetyl-FOXO1, nicotinamide (10 mM) and trichostatin A (500 nM) were added to the lysis buffer. When phospho-AMPK and phospho-ACC were analyzed, phosphatase inhibitor cocktail (Nakalai Tesk) were added to the lysis buffer. The lysates were then sonicated and centrifuged to remove insoluble matter. The protein concentration of the supernatant was measured using the Protein Quantification Kit-Rapid (Dojindo, Kumamoto, Japan). Supernatant fractions of equal protein concentration were analyzed by Western blotting as described previously (Tanno et al., 2010). The membrane was blocked with TBST (50 mM Tris-HCl, pH 7.5, 150 mM NaCl, 0.05% Tween 20) containing 5% non-fat milk or 5% BSA for 30 min. The membrane was incubated with primary antibodies overnight at 4°C. After washing with TBST, the membrane was incubated with a peroxidase-labeled secondary antibody (1∶10000 dilution) for 1 hour. The membranes were developed by standard methods using enhanced chemiluminescence.

### Reverse Transcription and Polymerase Chain Reaction

Total RNA was isolated using an RNeasy Mini Kit (Qiagen). First-strand cDNA was synthesized using SuperScript III (Invitrogen). DNA amplification was performed by RT-PCR using Taq DNA polymerase (New England Biolabs). The primer sequences used for RT-PCR were as follows: p53 forward 5′-AGTCACAGCACATGACGGAGGT-3′, reverse 5′-TACACATGTACTTGTAGTGGAT-3′; GAPDH forward 5′-ACCACAGTCCATGCCATCAC-3′, reverse 5′-TCCACCACCCTGTTGCTGTA; FOXO1 forward 5′-CAAAGTACACATACGGCCAATCC-3′, reverse 5′-TGCCTGGCTGCCATACGCTGGCA-3′; FOXO3a forward 5′-AACAGACCAGCCACCTTCTCTT -3′, reverse 5′-GCTGACAGAATTTGACAAGGCA -3′; FOXO4 forward 5′-ATACACCACCACCTCCTGCTGATG-3′, reverse 5′-CCAGATCCTGAGGCATTCTGTCAT-3′. GAPDH served as a control. For quantitative PCR, the cDNA amplification was performed in StepOne (Applied Biosystems, Foster City, CA) using TaqMan Universal Master Mix II, with UNG (Applied Biosystems) and TaqMan Gene Expression Assays for SOD2 (Mm00449726_m1) or β-actin (Mm01205647_g1). β-actin served as an internal control.

### Statistical Analysis

The data were expressed as means ± SEM. Comparisons between multiple groups were made by one-way ANOVA followed by a post-hoc Student-Neuman-Kuels test. Differences were considered significant if the P-value was less than 0.05. All analyses were carried out using SigmaStat (Systat).

## Results

### SIRT1 Modulators Affect the Intracellular ROS Levels and Cell Death Induced by Oxidative Stress

**Figure 1:**
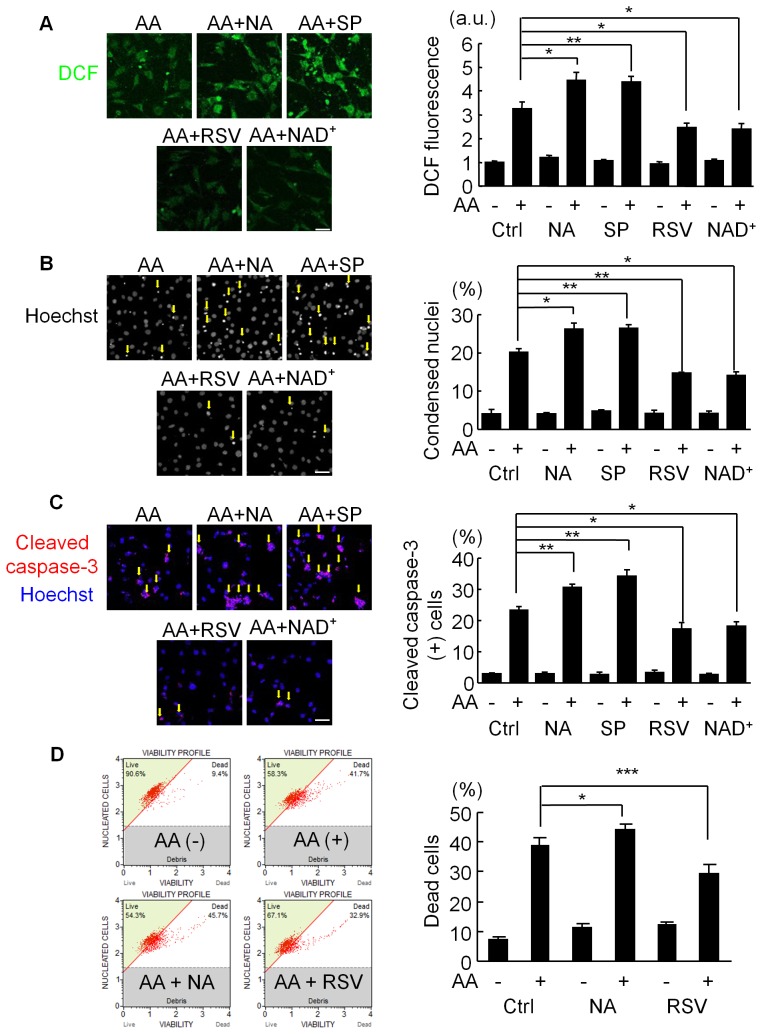
Effects of SIRT1 modulators on antimycin A-induced ROS generation and cell death. (A) Representative images (left) and quantitative analysis (right) of CM-H_2_DCFDA (DCF) fluorescence in C2C12 cells. Cells were treated with 200 µM antimycin A (AA) for 24 hours after their pretreatment with vehicle (Ctrl), 5 mM nicotinamide (NA), 60 µM splitomicin (SP), 10 µM resveratrol (RSV), or 1 mM NAD^+^ for 3 hours. Data are from three independent experiments. Scale bar: 10 µm. (B) (Left) Representative images of nuclear staining with Hoechst33342 in C2C12 cells treated as in (A). (Right) Quantification of apoptotic cells defined by nuclear condensation. Data are from three independent experiments. Scale bar: 10 µm. (C) (Left) Representative images of immunostaining for cleaved (active) caspase-3 in C2C12 cells treated as in (A). (Right) Quantification of cleaved caspase 3-positive cells. Data are from three independent experiments. Scale bar: 10 µm. (D) Viability profiles (left) and quantification of dead cells (right) in C2C12 cells treated for 9 hrs with 60 µM AA with or without pretreatment with 10 mM NA or 30 µM RSV (N = 7). ***p<0.001, **p<0.01, *p<0.05, n.s. = not significant. a.u. = arbitrary units.

We first analyzed the effect of SIRT1 modulators on the apoptosis induced by antimycin A in C2C12 cells. Antimycin A significantly elevated the intracellular ROS levels measured by CM-H_2_DCFDA staining (Figure 1A) and increased the number of apoptotic cells identified by nuclear condensation (Figure 1B) and activated caspase-3 (Figure 1C). Pretreatment with a SIRT1 inhibitor nicotinamide (NA) or splitomicin (SP) augmented antimicin A’s effects on both the increased ROS levels (Figure 1A) and apoptosis (Figure 1B and 1C), whereas pretreatment with the SIRT1 activator RSV or the SIRT1 cofactor NAD^+^ significantly decreased the ROS levels (Figure 1A) and inhibited the apoptosis (Figure 1B and 1C) induced by antimycin A. Dead cells were also analyzed by a Muse Cell Analyzer. NA augmented late apoptotic/necrotic cell death induced by antimycin A, whereas RSV decreased the cell death (Figure 1D).

**Figure 2:**
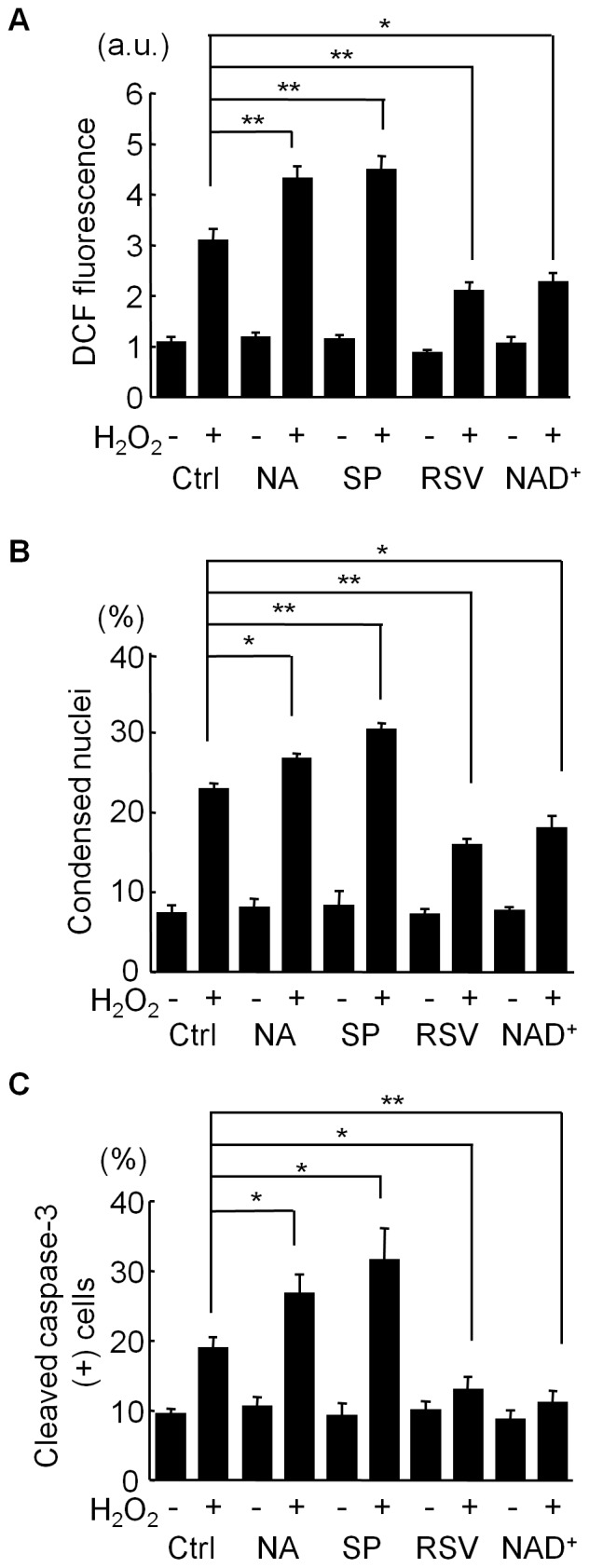
Effects of SIRT1 modulators on H_2_O_2_-induced ROS generation and apoptosis. C2C12 cells were treated with 50 µM H_2_O_2_ for 24 hours after their pretreatment with vehicle (Ctrl), 5 mM nicotinamide (NA), 60 µM splitomicin (SP), 10 µM resveratrol (RSV), or 1 mM NAD^+^ for 3 hours. (A) Intracellular levels of reactive oxygen species (ROS) were detected by CM-H_2_DCFDA (DCF). Data are from three independent experiments. (B and C) Cell death was analyzed by nuclear condensation (B), or by immunostaining for cleaved (active) caspase-3 (C). Data in each panel are from three independent experiments. **p<0.01, *p<0.05. a.u. = arbitrary unit.

Antimycin A releases a superoxide radical from mitochondria, which is dismutated into oxygen and hydrogen peroxide (H_2_O_2_) by endogenous superoxide dismutases (SODs). To examine whether SIRT1 modulators show similar anti-oxidative and anti-apoptotic effects on C2C12 cells treated with H_2_O_2_, we used H_2_O_2_ as an oxidant (Figure 2). Again, SIRT1 inhibitors increased the ROS levels (Figure 2A) and the number of apoptotic cells (Figure 2B and 2C), while SIRT1 activators significantly inhibited the H_2_O–induced increase in ROS levels (Figure 2A) and apoptosis (Figure 2B and 2C).

**Figure 3:**
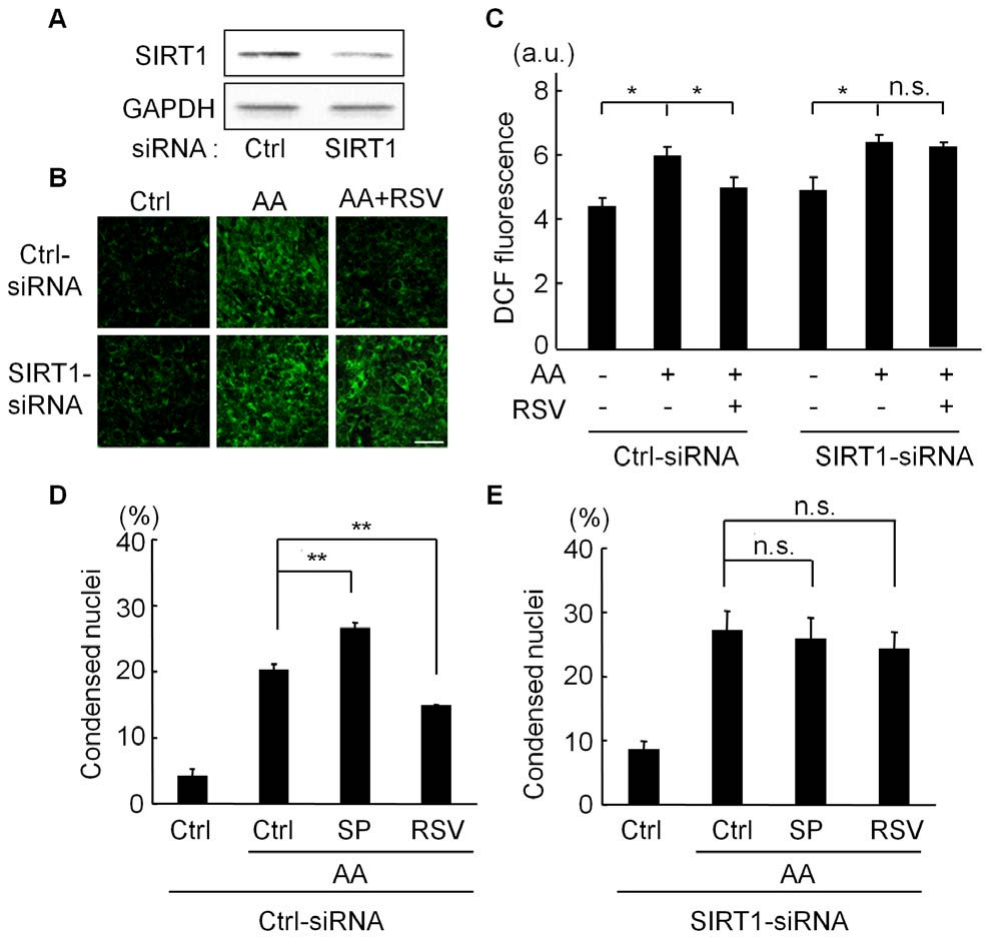
Effects of SIRT1 knockdown on functions of SIRT1 modulators. (A) Immunoblot analysis for SIRT1 in C2C12 cells transfected with control (Ctrl)- or SIRT1-siRNA. (B) Representative images of CM-H_2_DCFDA fluorescence. C2C12 cells transfected with control- (Ctrl-) or SIRT1-siRNA were treated with antimycin A (AA) (100 µM) for 3 hours after pretreatment with vehicle or resveratrol (RSV, 10 µM) for 6 hours. Scale bar: 10 µm. (C) Quantification of CM-H_2_DCFDA (DCF) fluorescence. (D and E) Quantification of cells with condensed nuclei. C2C12 cells transfected with control- (Ctrl-) or SIRT1-siRNA were treated with AA (200 µM) for 24 hours after pretreatment with vehicle (Ctrl), 10 µM RSV, or 60 µM splitomicin (SP) for 3 hours. Data are from three independent experiments. **p<0.01, *p<0.05. a.u. = arbitrary unit. n.s. = not significant.

To examine whether these SIRT1 modulators affect ROS levels and cell survival via SIRT1, we transfected C2C12 cells with SIRT1-siRNA. SIRT1 knockdown by siRNA (Figure 3A) abolished RSV’s anti-oxidative (Figure 3B and 3C) and anti-apoptotic functions (Figure 3D and 3E). In the presence of SIRT1-siRNA, the antimycin A-induced apoptosis increased to the level seen in SP-treated control-siRNA cells (Figure 3D and 3E), and SP did not further enhance the apoptosis in cells transfected with SIRT1-siRNA (Figure 3D and 3E). These results indicate that the SIRT1 modulators affected the cellular ROS levels and apoptosis via SIRT1 in C2C12 cells.

### SIRT1 Modulators Affect Both p53-dependent and p53-independent Apoptosis Induced by Antimycin A

**Figure 4:**
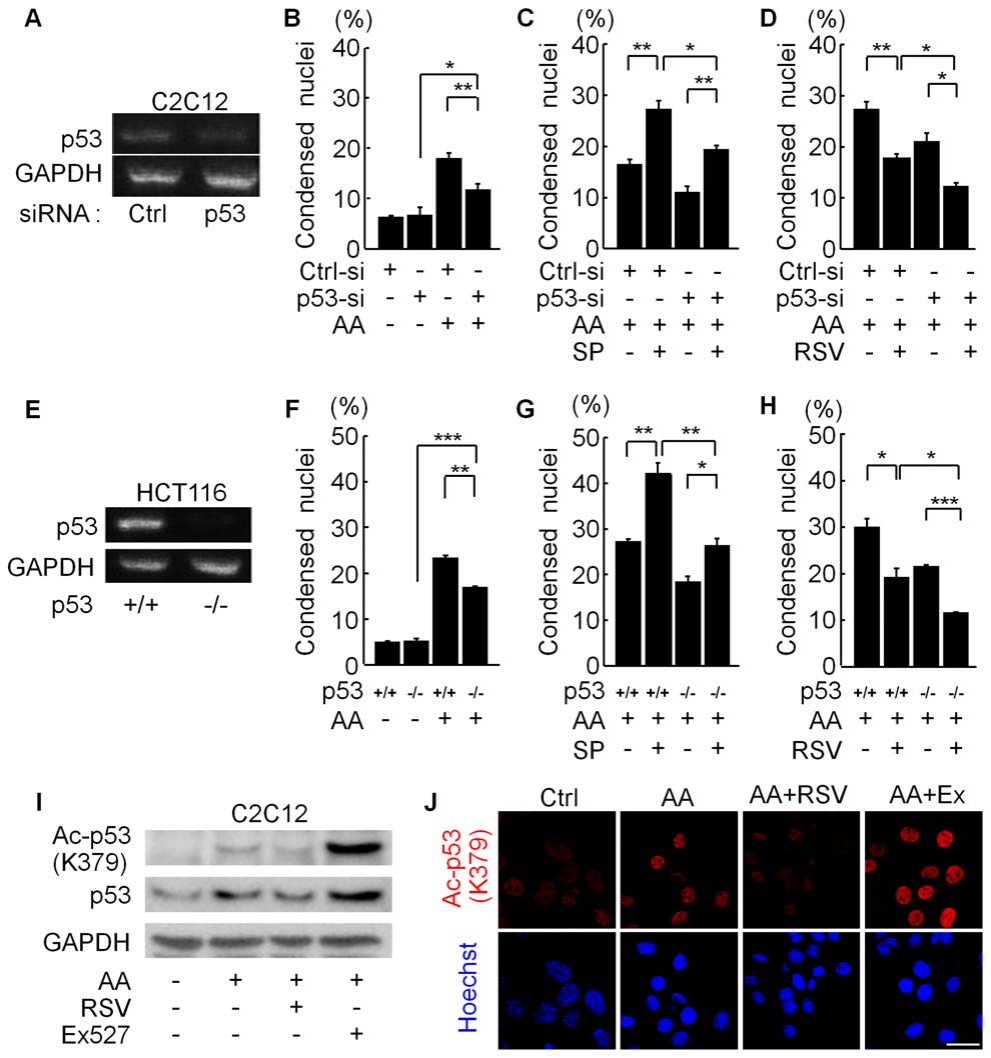
Effects of p53 knockdown on the functions of SIRT1 modulators. (A) RT-PCR analysis for p53 and glyceraldehyde 3-phosphate dehydrogenase (GAPDH) in C2C12 cells transfected with siRNA against negative control (Ctrl) or p53. (B) C2C12 cells transfected with control- (Ctrl-si) or p53-siRNA (p53-si) were treated with vehicle or AA (50 µM) for 24 hours. Apoptotic cells with condensed nuclei were quantified. Data are from three independent experiments. (C and D) The percentage of nuclear-condensed C2C12 cells was analyzed. C2C12 cells transfected with control- (Ctrl-si) or p53-siRNA (p53-si) were pretreated with vehicle or either 60 µM splitomicin (SP in C) or 10 µM RSV in D for 3 hours followed by incubation with 50 µM antimycin A (AA) for 24 hours. Data are from three independent experiments. (E) RT-PCR analysis for p53 mRNA in wild type (p53+/+) or p53-deficient (p53−/−) HCT116 cells. (F) Quantification of apoptotic cells with nuclear condensation in wild-type (p53+/+) or p53-null (p53−/−) HCT116 cells treated with vehicle or AA for 24 hours. Data are from three independent experiments. (G and H) Analysis of apoptosis in HCT116 cells treated with SIRT1 modulators. Wild-type (p53+/+) or p53-deficient (p53−/−) HCT116 cells were pretreated with 60 µM SP (G) or 10 µM RSV (H) for 3 hours and then incubated with 50 µM AA for 24 hours. Data are from three independent experiments. ***p<0.001, **p<0.01, *p<0.05. (I) Representative immunoblots of acetyl-p53 (K379) and p53 in C2C12 cells treated with vehicle or antimycin A (AA, 50 µM) with or without pretreatment with resveratrol (RSV) or Ex527. All cells were treated in the presence of 50 nM of trichostatin A. (J) Representative images of immunostaining for acetyl-p53 (Lys379) in C2C12 cells treated as in A. Scale bar: 20 µm.

The transcription factor p53 is deacetylated and inactivated by SIRT1 [7], [8]. Since antimycin A activates p53 [19], we examined whether p53 mediates the apoptosis induced by antimycin A in C2C12 cells. p53 knockdown itself did not affect cell death in vehicle-treated C2C12 cells (Figure 4A and 4B). In the presence of antimycin A, however, p53 knockdown significantly decreased the number of apoptotic cells but failed to completely suppress apoptosis (Figure 4B), indicating that both p53-dependent and p53-independent mechanisms regulated the antimycin A-induced apoptosis of C2C12 cells. Transfection with p53-siRNA partially inhibited the apoptosis-promoting activity of the SIRT1 inhibitor SP (Figure 4C), suggesting that the p53-dependent pathway is involved in the SP-induced enhancement of apoptosis. However, SP still enhanced the antimycin A-induced apoptosis in p53-knockdown cells (Figure 4C). Furthermore, in the presence of p53-siRNA, RSV still reduced the number of apoptotic cells (Figure 4D).

To exclude the possibility that residual p53 contributed to the apoptosis in cells transfected with p53-siRNA, we examined p53-positive (p53 (+/+)) and p53-deficient (p53 (−/−)) HCT116 human colon carcinoma cells (Figure 4E). The number of antimycin A-induced apoptotic p53 (−/−) cells was significantly smaller than that of p53 (+/+) cells, indicating that the p53 pathway is involved in the cell death induced by antimycin A in HCT116 cells (Figure 4F). SP and RSV significantly increased and suppressed the apoptosis of p53 (−/−) cells by antimycin A, respectively (Figure 4G and 4H), although the number of apoptotic cells increased by SP treatment was significantly lower in the p53 (−/−) cells than that in the p53 (+/+) cells (Figure 4G). These findings indicate that both p53-dependent and p53-independent mechanisms regulated the apoptosis triggered by oxidative stress in C2C12 cells and HCT116 cells, and that SIRT1 modulators affected both mechanisms to regulate cell death.

Since SIRT1 inhibits p53 activity by promoting p53 deacetylation [7], [8], we examined effects of SIRT1 modulators on p53 acetylation level. Western blotting and immunocytochemistry showed that antimycin A increased acetyl-p53 level. Treatment of cells with RSV reduced acetyl-p53 level, whereas a specific SIRT1 inhibitor Ex527 strongly augmented p53 acetylation (Figure 4I and 4J).

### FOXO1, FOXO3a, and FOXO4 are Involved in the Resveratrol-dependent Induction of SOD2

**Figure 5:**
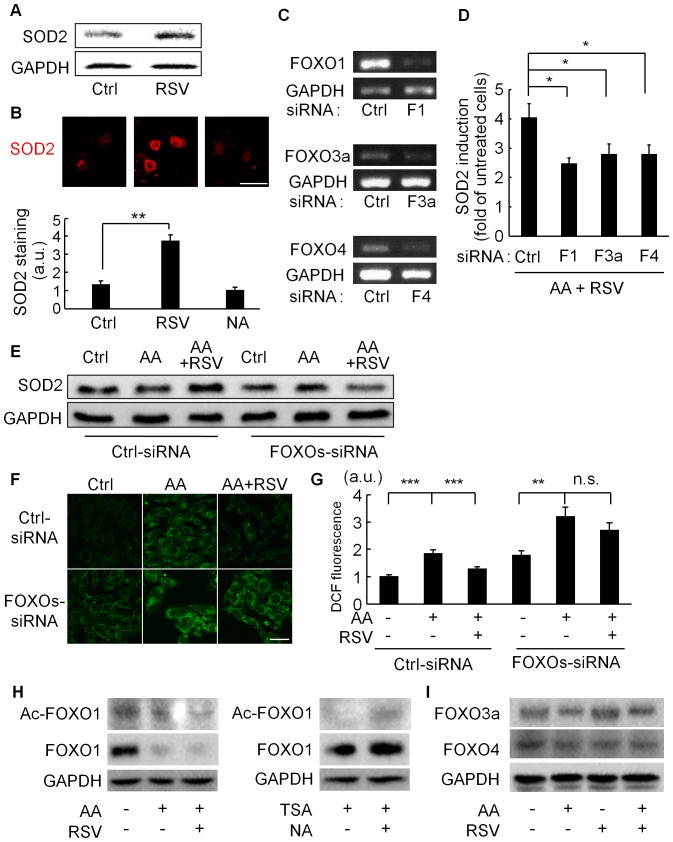
Resveratrol up-regulates SOD2 and suppresses antimycin A-induced ROS via FOXOs. (A) C2C12 cells were incubated in the presence or absence of RSV (100 µM) for 6 hours followed by western blotting to analyze the SOD2 protein level. (B) Representative images for SOD2 immunostaining in cultured neonatal rat ventricular myocytes (upper) and quantified data (lower). Cells were incubated in the presence of vehicle (Ctrl), resveratrol (RSV), or nicotinamide (NA) for 48 hours. Scale bar: 10 µm. (C) RT-PCR analyses for FOXO transcription factors in C2C12 cells transfected with negative-control siRNA (Ctrl), or siRNA against FOXO1 (F1), FOXO3a (F3a), or FOXO4 (F4). (D) Quantitative analysis of SOD2 mRNA by real-time RT-PCR in C2C12 cells (N = 6). After being transfected with siRNAs against negative-control (Ctrl), FOXO1 (F1), FOXO3a (F3a), or FOXO4 (F4), the cells were pretreated with resveratrol (RSV, 30 µM) for 3 hours, then incubated with antimycin A (AA, 50 µM) for 24 hours. (E) Immunoblot analysis for SOD2. C2C12 cells transfected with control-siRNA (Ctrl-siRNA) or siRNAs against all three FOXOs (FOXO1, FOXO3a, FOXO4) were preincubated with vehicle or RSV (30 µM) for 3 hours followed by treatment with AA (50 µM) without serum for 24 hours. Total-cell lysates were subjected to immunoblot analysis. (F) Representative images of CM-H_2_DCFDA-stained C2C12 cells. Cells transfected with control-siRNA or a mixture of all three FOXO siRNAs (FOXOs-siRNA) were treated with AA (200 µM) for 24 hours after pretreatment with vehicle or RSV (30 µM) for 3 hours. Scale bar: 10 µm. (G) Quantification of CM-H_2_DCFDA (DCF) fluorescence. Data are from three independent experiments. (H) Immunoblots for acetyl-FOXO1 and FOXO1 in C2C12 cells. Cells were treated with vehicle, antimycin A (AA, 60 µM), or RSV (30 µM)+AA (left panel), or were incubated with vehicle or nicotinamide (NA, 20 mM) in the presence of trichostatin A (TSA) (right panel). (I) Representative immunoblots for FOXO3a and FOXO4 in C2C12 cells treated with vehicle or AA with or without RSV. ***p<0.001, **p<0.01. n.s. = not significant. a.u. = arbitrary units.

We next examined whether FOXO transcription factors played a role in the p53-independent regulation of the antimycin A-induced apoptosis by SIRT1 modulators, especially RSV. FOXOs have dual roles in response to oxidative stress, in which they both promote apoptosis and induce ROS-detoxifying enzymes such as SOD2. As previously reported [13], [16], RSV induced SOD2 expression in C2C12 cells and neonatal rat cardiomyocytes (Figure 5A and 5B). In mammals, the FOXO family consists of FOXO1, FOXO3a, FOXO4, and FOXO6. FOXO1, FOXO3a, and FOXO4 are widely expressed in various tissues, whereas FOXO6 is predominantly expressed in the brain [20]. We therefore analyzed FOXO1, FOXO3a, and FOXO4 in the following experiments. Because all three of these FOXOs were expressed in C2C12 cells, we examined which ones are involved in SOD2’s induction by RSV under antimycin A treatment, by using siRNAs for each FOXO (Figure 5C). We found that the individual knockdown of FOXO1, FOXO3a, or FOXO4 significantly reduced the expression of SOD2 (Figure 5C and 5D). When the C2C12 cells were transfected with a mixture of siRNAs (FOXOs-siRNA), against FOXO1, FOXO3a, and FOXO4, SOD2’s induction by RSV was abolished (Figure 5E). Furthermore, monitoring the intracellular ROS levels by CM-H_2_DCFDA showed that the decrease in ROS levels by RSV was blocked by the FOXOs-siRNA (Figure 5F and 5G).

Because SIRT1 regulates FOXO activity via dacetylation, we assessed the effect of RSV on acetyl-FOXO1 level (Figure 5H). In the presence of antimycin A, RSV significantly reduced acetyl-FOXO1 level (Figure 5H), indicating that RSV enhanced the deacetylation of FOXO1. SIRT1 inhibitor NA increased acetyl-FOXO1 level (Figure 5H). Unexpectedly, we found that treatment of C2C12 cells with antimycin A decreased FOXO1 protein level, which was not affected by RSV. FOXO3a and FOXO4 protein levels were also downregulated by antimycin A treatment (Figure 5I). Down regulation of FOXO proteins by antimycin A made us difficult to detect acetylated FOXOs by immunoprecipitation experiments (data not shown).

### Knockdown of FOXO1, FOXO3a, and FOXO4 Completely Inhibited the Cell-protective Function of Resveratrol in C2C12 Cells

**Figure 6:**
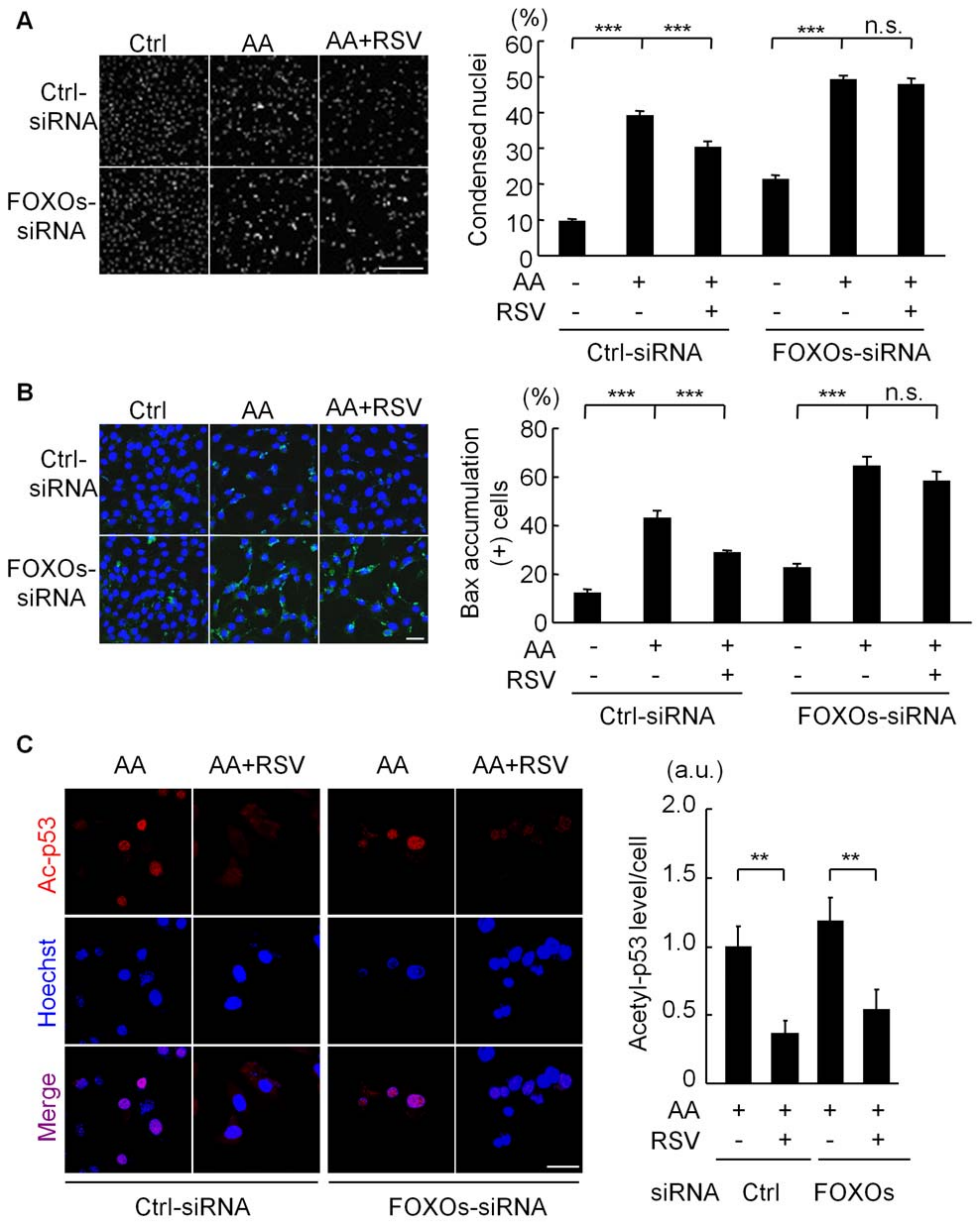
FOXOs mediate the anti-apoptotic effect of resveratrol. (A) (Left) Representative images of nuclear staining with Hoechst33342 in C2C12 cells. Cells transfected with control-siRNA (Ctrl-siRNA) or a mixture of siRNAs against all three FOXOs (FOXO1, FOXO3a, FOXO4) (FOXOs-siRNA) were pretreated with vehicle or resveratrol (RSV, 30 µM) for 3 hours and then incubated with antimycin A (AA, 50 µM) for 24 hours under serum-free conditions. Scale bar: 50 µm. (Right) Quantification of apoptotic cells defined by nuclear condensation. Data are from three independent experiments. (B) (Left) Representative images of immunostaining for BAX (green) in C2C12 cells treated as in A. Scale bar: 10 µm. (Right) Quantification of cells with BAX accumulation. Data are from three independent experiments. (C) Representative immunofluorescence images for acetylated p53 (red) in C2C12 cells treated as in A. The acetylation of p53 was detected by immunostaining using an antibody against acetyl-p53. Data are from three independent experiments. Scale bar: 20 µm. ***p<0.001, **p<0.01, n.s. = not significant.

Finally, we examined whether the knockdown of all three FOXO mRNAs affects RSV’s cytoprotective function in C2C12 cells treated with antimycin A. RSV significantly inhibited the cell death induced by antimycin A in C2C12 cells transfected with control-siRNAs, whereas its cytoprotective effect was completely abolished by the expression of the three FOXO-siRNAs (Figure 6A). The antimycin A-induced accumulation of BAX, an early-stage indicator of apoptosis, was also inhibited by RSV pretreatment of C2C12 cells transfected with control-siRNA (Figure 6B). This suppression of BAX accumulation by RSV was inhibited by knockdown of the three FOXO mRNAs (Figure 6B). In addition, the FOXOs-siRNA significantly increased the number of antimycin A-induced apoptotic cells, compared with cells transfected with control-siRNA (Figure 6A and 6B). These findings indicate that the FOXOs were indispensable for the cellular protective mechanism elicited by RSV against oxidative stress. To exclude the possibility that the FOXOs-siRNAs affected the deacetylation status of p53, we immunostained C2C12 cells for acetyl-p53. RSV significantly reduced the acetyl-p53 levels in C2C12 cells transfected with both the control-siRNA and FOXOs-siRNAs (Figure 6C), indicating that the knockdown of FOXOs did not affect the acetylation levels of p53.

### RSV did not Modulate AMPK Activity in C2C12 Cells

**Figure 7:**
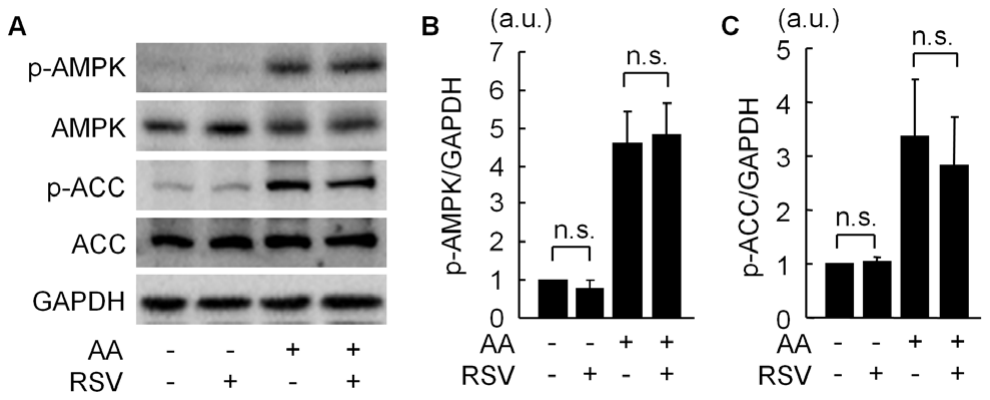
The effect of resveratrol on AMPK activity in C2C12 cells. (A) Representative immunoblots of phospho-AMPK (Thr172), total AMPK, phospho-acetyl-CoA carboxylase (ACC) (Ser79), total ACC, and GAPDH in C2C12 cells treated with vehicle or 60 µM antimycin A (AA) with or without 30 µM resveratrol (RSV). (B) Quantification of phospho-AMPK (Thr172) level normalized to GAPDH (N = 5). (C) Quantification of phospho-ACC (Ser79) level normalized to GAPDH (N = 5). n.s. = not significant.

Recent reports have demonstrated that RSV activates AMPK, which contributes to protective effects of RSV [21], [22]. We examined whether RSV modulates AMPK activity in our model. Activated AMPK was monitored by levels of phosphorylated AMPKα and phosphorylated acetyl-CoA carboxylase (ACC), a downstream enzyme of AMPK. Neither phospho-AMPKα nor phospho-ACC was changed by RSV treatment alone. Antimycin A markedly induced AMPKα and ACC phosphorylation, which was not promoted by RSV (Figure 7A and 7B). These findings indicate that RSV did not activate AMPK in our experimental setting.

## Discussion

In this study we examined whether p53 and/or FOXOs participate in the effect of SIRT1 modulators in C2C12 cells exposed to a high level of oxidative stress. Antimycin A was used to induce and liberate ROS from mitochondria. Although the modulation of p53 by SIRT1 partially inhibited the oxidative stress-induced cell death, the knockdown of FOXO1, FOXO3a, and FOXO4 completely abolished the cell-protective effect of the SIRT1 activator RSV. Moreover, unexpectedly, we found that FOXO1, FOXO3a, and FOXO4 are essential for cell survival under conditions of high oxidative stress.

### Cell Survival via the Modulation of FOXOs Activity

In mammals, there are four FOXO transcription factor members, whose functions appear to overlap with each other [10]. In fact, here we observed that SOD2’s induction by RSV in the presence of antimycin A was significantly inhibited by the individual knockdown of FOXO1, FOXO3a, or FOXO4 (Figure 5D). Under oxidative stress conditions, the SIRT1 activator RSV increased the SOD2 level (Figure 5E), reduced the ROS levels (Figure 5F and 5G), and promoted cell survival (Figure 6A and 6B) via the FOXOs.

We previously showed that SOD2 knockdown completely inhibits RSV’s anti-apoptotic function against antimycin A [13]. Taken together, the results indicate that the increase in SOD2 expression by RSV under antimycin A-induced oxidative stress is necessary for cell survival. The knockdown of FOXO1, FOXO3a, and FOXO4 inhibited SOD2’s induction by RSV, but failed to abolish the baseline expression of SOD2 (Figure 5E). This may indicate that the upregulation of ROS-detoxifying enzymes including SOD2 is important for RSV’s cell-protective roles against high ROS levels. SOD2 catalyzes the dismutation of O_2_^−^ to H_2_O_2_, which is further metabolized to H_2_O and O_2_ by catalase and peroxidases. Because FOXOs can induce catalase and some peroxidases [9], [23] and because SIRT1 modulators exerted similar effects on H_2_O_2_-induced cell death as on antimycin A-induced apoptosis (Figures 1 and 2), RSV’s cell-survival effect via FOXOs depends not only on SOD2’s induction but also on the increase in downstream ROS-detoxifying enzymes.

In the absence of antimycin A, the expression of the three FOXO-siRNAs unexpectedly increased the cellular ROS levels and the number of apoptotic cells, compared with cells transfected with control-siRNA (Figures 5F and 6). Thus, in the absence of FOXOs, the ROS produced by cellular metabolism, such as mitochondrial oxidative phosphorylation, may trigger apoptosis, and FOXOs may constantly protect cells by reducing the cellular ROS levels.

The FOXOs’ activity is modulated by acetylation and deacetylation [9]. Whereas SIRT1 activation enhances the transcriptional activity of FOXO1 [24], FOXO3a [25], and FOXO4 [26] on ROS-detoxifying enzymes by deacetylation, the CBP/p300-mediated acetylation of FOXOs impairs their DNA-binding activity [9]. We recently showed that the deacetylation of p300 by SIRT1 promotes p300’s ubiquitination and downregulation, and that RSV reduces the p300 protein level [15]. Thus, p300’s downregulation by SIRT1 could also increase the deacetylated FOXOs levels. In addition, RSV is reported to promote FOXO1’s nuclear retention via SIRT1-mediated deacetylation [27], which may increase its transcriptional activity.

### Cell Survival via the Modification of p53 Activity

We showed here that p53 knockdown in C2C12 cells and p53-deficient HCT 116 cells exhibited less antimycin A-induced apoptosis than the parental C2C12 cells and p53-positive HCT116 cells, respectively (Figure 4B and 4F), indicating that p53 was involved in the apoptotic pathway induced by oxidative stress. Under oxidative stress, there were more apoptotic cells in the presence of the SIRT1 inhibitor SP in p53-positive C2C12 cells and p53 (+/+) cells than in p53-knockdown C2C12 cells and p53 (−/−) HCT116 cells, respectively (Figure 4C and 4G). These results indicate that the p53-dependent apoptotic mechanism is also regulated by SIRT1 modulators, which is consistent with SIRT1’s inhibitory effect on p53’s activity under ionizing radiation, etoposide, and H_2_O_2_ treatment [7], [8].

### SIRT1 Inhibitors as Inducers of Apoptosis

We showed here that the inhibition of SIRT1 enhances the apoptosis elicited by oxidative stress (Figure 1 and 2). Although SIRT1 regulates both p53-dependent cell death and FOXOs-dependent cell survival signals under oxidative stress conditions, our findings suggest that the FOXOs-dependent cell-protective mechanism, which induces the expression of ROS-detoxifying enzymes, is more important for the cell-fate decision. More than 50% of human tumors contain a mutation or deficiency in p53 [28]. Our results suggest that SIRT1 inhibition enhances the cell-death effect of ROS-generating anti-cancer drugs, even in p53-deficient or p53-mutated cancer cells. Thus, SIRT1 inhibition may contribute to the treatment of cancer patients in combination with ROS-producing cancer therapies such as ionizing radiation and alkylating antineoplastic agents. In addition, FOXO inhibitors may be alternative agents for enhancing cancer chemotherapy.
